# Exploring the experiences of stroke patients treated with transcranial magnetic stimulation for upper limb recovery: a qualitative study

**DOI:** 10.1186/s12883-020-01936-5

**Published:** 2020-10-06

**Authors:** Eline CC van Lieshout, Lilliane D Jacobs, Maike Pelsma, Rick M Dijkhuizen, Johanna MA Visser-Meily

**Affiliations:** 1 Biomedical MR Imaging and Spectroscopy Group, Center for Image Sciences, University Medical Center Utrecht and Utrecht University, Utrecht, The Netherlands; 2 Center of Excellence for Rehabilitation Medicine, UMC Utrecht Brain Center, University Medical Center Utrecht and Utrecht University, and De Hoogstraat Rehabilitation, Utrecht, The Netherlands; 3 Department of Rehabilitation, Physical Therapy Science and Sports, UMC Utrecht Brain Center, University Medical Center Utrecht and Utrecht University, Heidelberglaan 100, Utrecht, CX 3584 the Netherlands

**Keywords:** Stroke, Non-invasive brain stimulation, TMS, Qualitative study, Upper limb

## Abstract

**Background:**

Transcranial magnetic stimulation (TMS) treatments have shown promise in improving arm recovery in stroke patients. Currently, little is known about patients’ experiences with repetitive TMS treatment, and this lack of knowledge may affect optimal implementation in clinical practice. The aim of this explorative study was to gain insight in the perceived effects and experiences of the design and delivery of a rTMS treatment for upper limb recovery from the perspectives of stroke patients.

**Methods:**

This qualitative study was conducted as part of a randomized controlled trial (RCT) in a specialized rehabilitation center. Data were collected through face-to-face semi-structured interviews with 13 stroke patients who completed a 10-day rTMS intervention for upper limb recovery. The interviews were recorded, transcribed verbatim and analyzed using thematic analysis.

**Results:**

The major themes that emerged from the patients’ feedback were the following: positive experiences of the treatment (experienced physical effects, comfort, therapeutic relationship, receiving information, learning about the brain, no burden of added rTMS treatment session, no unpleasant aspects), concerns (effects of stimulation of the brain, equipment, logistics), general experience of recovery, experienced psychological effects (grateful, sense of purpose, recovery as extra motivation to exercise, disappointment and hope of group allocation), and motivation to participate (personal benefit and cognitions, altruism). Important components related to the positive experience of the design and delivery of the treatment included comfort (i.e. moment of relaxation) and the sensation of a painless treatment without side-effects. Key concerns included uncertainty and anxiety about possible negative consequences and group allocation.

**Conclusions:**

This study demonstrates that rTMS is well accepted by stroke patients with an upper limb paresis. Besides the expectation of a therapeutic benefit, the patients reported various psychological effects. Positive experiences, such as the provision of a short moment of relaxation each day, could have practical implications for clinical stroke rehabilitation settings aimed at improving patient satisfaction. Explanation about and feedback from routine motor recovery progression monitoring at fixed times post-stroke is also valued by patients. Negative emotions may be limited or avoided by transparent and recurrent information delivery in future trials.

## Background

Stroke is one of the leading causes of disability and can have severe consequences for upper limb function [1]. Patients with impaired upper limb function often experience limitations in activities, and restrictions in participation, with a consequent decline in health-related quality of life [2, 3]. While less than 50% of stroke patients without initial hand capacity on admission in a rehabilitation center regains some hand capacity at discharge, more than 75% of patients with residual hand capacity regain advanced hand capacity at discharge [4]. Thus, many patients experience at least some degree of recovery of their lost motor function over time [5]. Restorative therapies that lead to a full return of all behaviors as before injury are currently under study [6, 7]. Systematic reviews and meta-analyses of studies performed in the acute to chronic phases post-stroke have shown that non-invasive brain stimulation (NIBS) can induce therapeutic effects on upper limb function [6–8]. Very recently, level A evidence (definite efficacy) has been indicated for low-frequency rTMS of the contralesional primary motor cortex (M1) in the post-acute stage (1 week to 2 months post-stroke) for improvement of upper limb motor function in stroke patients [9].

NIBS techniques, such as repetitive transcranial magnetic stimulation (rTMS) and transcranial direct current stimulation (tDCS), have the potential to increase or decrease cortical excitability, dependent on the parameters of stimulation [10–12]. In a multi-institutional study, only transient side effects, namely minor dizziness, discomfort at stimulation site, and mild headache, were reported after rTMS by 22 out of 1725 patients [13]. This confirms the good tolerance for this type of treatment. An interview study that explored the views and experiences of 21 patients who underwent tDCS combined with robotic therapy for upper limp recovery, revealed that the therapy was generally experienced as effective and comfortable [14]. However, some patients also reported discomfort (e.g. painful, itchy stimulation) or feelings of uncertainty about the consequences of tDCS. So far, the subjective experiences of patients receiving rTMS have not been assessed yet. Insights from the patients’ perspectives may help researchers and health care professionals to identify topics that are important for patients undergoing a treatment, which could improve future trial design and subsequent clinical implementation. In addition, patients’ expectations can provide important predictors of treatment outcomes [15–17].

We conducted a qualitative study that ran parallel to a randomized controlled trial (B-STARS), in which stroke patients undergo cTBS (continuous theta burst stimulation), a variant of rTMS, within the first month post-stroke to promote upper limb recovery [18]. cTBS can decrease cortical excitability and consists of an uninterrupted train of 20 to 40 s of TBS. [19] The treatment starts within 21 days after stroke onset, because the brain may be most responsive to neurorehabilitation in this time-window, and is always followed by upper limb training, which is part of inpatient rehabilitation care. The design and methods of the RCT have been described in detail in a protocol article [18]. The objectives of the current qualitative study were to identify and understand the patients’ perceived effects and experiences of the design and delivery of a TMS-based treatment for upper limb recovery. Results from this study could reveal aspects that interfere with or promote effective clinical implementation of rTMS treatment and lead to improvement of future rTMS trials.

## Methods

### Design

Semi-structured interviews were conducted with a subset of patients who participated in the B-STARS (Brain-Stimulation for Arm Recovery after Stroke) trial [18]. The B-STARS trial is a stratified randomised controlled trial to investigate the effects of rTMS (cTBS) on arm recovery after stroke. The B-STARS trial is still ongoing, and expected to be completed by 2020/2021. The B-STARS is registered with the study number NL5952, November 28, 2016.

#### B-STARS intervention

The included patients were randomly allocated to two groups: 1) real or 2) sham cTBS of the contralesional primary motor cortex. Patients underwent 10 treatment sessions in the morning or afternoon, followed by usual care upper limb training, over a period of 2 weeks during their inpatient rehabilitation period. A treatment session had an average duration of ten minutes. Upper limb therapy started 5 min after cTBS and consisted of 60 min of upper limb exercises individualized to each patient, delivered by experienced physical therapists. A Neuro-MS/D Advanced Therapeutic stimulator (Neurosoft, Russia) in combination with a 70 mm figure-of-eight coil was used for cTBS. Sham stimulation was administered with the protocol in sham mode (generating pulses at 90% lower intensity of the RMT). All patients were naïve to rTMS and therefore should not be able to distinguish between real and sham rTMS. Full details of the B-STARS trial protocol have been described elsewhere [18]. The study protocol was approved by the Medical Ethics Review Committee of the University Medical Center Utrecht and the participating rehabilitation center. The study was registered in the Dutch Trial Register (NL5952). This study conforms to the consolidated criteria for reporting qualitative research (COREQ) guidelines (Additional file 1).

### Participants

Patients met the in- and exclusion criteria of the B-STARS trial [18]: a first-ever ischemic or hemorrhagic stroke, ≥18 years, ≤21 days post-stroke, a paresis of one arm (Motricity Index score ≥ 9 on shoulder abduction) and the ability to provide informed consent. Patients were excluded if they had a disabling medical history (severe head trauma, severe or recent heart disease, coercively treated at a psychiatric ward), history of epilepsy, normal to almost normal use of the hand (Motricity Index score of 33 on pinch grip [20]), severe deficits in communication, memory, or understanding that impede proper study participation, or contra-indications for TMS and MRI. Participants of the B-STARS trial who completed the intervention period were asked to participate by telephone or face-to-face in the qualitative study by EvL or LJ. Participants who were willing to be interviewed were approached for an appointment. Written informed consent had already been obtained for the randomized controlled trial, in which patients could indicate whether they could be approached for the qualitative study after completion of the intervention period.

### Data collection

Patients who took part and completed the rTMS intervention period were asked to be interviewed. To achieve heterogeneity in the sample, we invited patients who completed the trial, as well as consecutively patients who were still in the trial for follow-up measurements. Patients from the real as well as the sham cTBS group were included in order to get a complete picture of the experiences of patients undergoing the treatment. Identification of group differences was not the aim of this paper. The semi-structured interviews were undertaken on a single occasion between May and October 2019. Participants were given the choice of location of the interview: at the rehabilitation center or at the patient’s home. The semi-structured interviews were conducted by a female psychologist (LJ) who was not involved in the recruitment and data collection process and who was blinded for the patient’s group allocation. Patients were informed about the professional background of the interviewer and the purpose of the interviews. The interviewer was trained in interview techniques before the start of the study. The duration of each interview was between 30 and 60 min. The interviews were conducted in Dutch. All interviews were audio-recorded, and field notes about the patient’s behaviour and contextual matters were taken during the interview. The interviewer used a topic guide focused on (the motivation for) participation in the treatment; emotions, cognitions and sensations before, during and after the treatment; effect of the treatment on arm function; and expectations in relation to rTMS (for a complete topic guide, see Additional file 2). The interviewer briefly explained the purpose of the interview, and techniques as hemming, summarizing and reflecting were used.

### Data analysis

All the audio recordings were transcribed verbatim. The transcripts were crosschecked by the first author (EvL). Confidential information, including health care services, were deleted from the transcripts. Qualitative data analysis was conducted by the first (EvL) and second author (LJ). The first author, female psychologist and researcher, knew the patients from the RCT, therefore, involving an external researcher (LJ) to the study minimized risk of bias. The process of data collection and analysis was iterative. Thematic analysis was used to interpret the data, which involved reading and rereading the responses from all interviewed patients and generating initial codes, independently by the two researchers (EvL and LJ). Thematic analysis was performed according to the six phases as described by Braun and Clarke [21]. To confirm correct application of the six phases of Braun and Clarke, the 15-point checklist was used (Additional file 3). Recurrent themes were identified and listed. The researchers reviewed, compared and discussed the (emerging) themes until the final themes could be determined. In addition, throughout the analysing process, data saturation was discussed. Data saturation was accomplished when no new themes were added during the last three interviews [22]. Three patients provided feedback on the findings. Appropriate quotes and citations were selected to illustrate each theme. MAXQDA 2018 (VERBI Software, 2017) was used for data analysis.

## Results

Thirteen patients with a mean age of 56.5 (SD 13.0; range 32–77 years) were interviewed. We excluded one patient because his memory deficits gave serious problems during the interview. All but three patients who were approached for an interview agreed to participate. Of these three patients, two could not participate due to logistic reasons and one felt uncomfortable with an interview because of aphasia. The majority of the patients were women (61.5%). The time post-stroke varied between 1 and 25 months. 61.5% had a right-sided hemiparesis. Characteristics of the patients are listed in Table 1.

**Table 1:** Demographic characteristics of patients

Patient ID	Gender (M/F)	Age (years)	Time since stroke at interview (months)	Educational level^a^	Side of hemiparesis (L/R)	Stroke severity score at hospital admission^b^	Independence in ADL at rehabilitation admission^c^	Screening for anxiety and depression^d^	Level of upper limb impairment post-treatment^e^
Stimulated group									
1	M	40–50	2	Medium	R	12	20	6	65
2	F	50–60	3	Low	L	6	12	11	64
3	F	40–50	2	Medium	L	10	16	11	28
4	F	50–60	13	Low	R	7	12	3	9
5	F	50–60	12	Low	R	6	12	7	62
Sham group									
6	M	70–80	4	Low	L	5	4	13	9
7	M	70–80	3	Low	R	6	6	0	30
8	F	50–60	2	High	R	12	11	6	56
9	F	60–70	9	Low	L	11	17	5	54
10	F	40–50	15	High	R	–	8	3	65
11	F	50–60	10	High	L	15	9	3	10
12	M	30–40	25	High	R	21	20	8	49
13	M	70–80	1	High	R	6	15	7	56

^a^Educational level: low = did not complete secondary school or completed low level secondary school; medium = completed medium level secondary school; high = completed upper level secondary school and/or university degree; ^b^Based on National Institutes of Health Stroke Scale (0–42, higher scores indicating more severe stroke) [23]; ^c^Based on Barthel Index (0–20, higher scores indicating greater independence [24]; ^d^Based on Hospital Anxiety and Depression Scale (0–21); higher scores indicating higher risk of anxiety and/or depression disorders [25]; ^e^Based on Fugl-Meyer Assessment upper extremity (0–66, higher scores indicating better performance, 1 week post-treatment, max. 6 weeks post-stroke) [26]

The results are presented in two main sections. The first section describes the patient’s experiences with the treatment and presents the themes positive experiences with the treatment and concerns. During the interviews the patients also shared experiences about participation in an RCT, which are outlined in the themes experienced psychological effects and motivation to participate. An overview of the themes and subthemes is given in Table 2. Patient’s quotations are shown in italics, and interpretation of the patient’s words is presented alongside the quotations.

**Table 2:** Overview themes and subthemes

Patient experiences with the treatment	Subthemes
Positive experiences of the treatment	Experienced physical effects
	Comfort
	Therapeutic relationship
	Receiving information
	Learning about the brain
	No burden of added TMS treatment session
	No unpleasant aspects
Concerns	Effects of stimulation of the brain
	Equipment (chair and coil)
	Logistics
Participation in an RCT	
General experience of recovery	
Experienced psychological effects	Grateful
	Sense of purpose
	Recovery as extra motivation to exercise
	Disappointment and hope of group allocation
Motivation to participate	Personal benefit and cognitions
	Altruism

### Patient’s experiences with the treatment

#### Positive experiences of the treatment

In general, patients had positive experiences receiving the rTMS treatment. Several patients mentioned that undergoing the treatment sessions felt as a special moment during their day: *“For me it was… it sounds very silly… a getaway within my rehabilitation.” (P5)* In addition, a good vibe during the treatment sessions was emphasized by patients.

All patients would recommend participation in the intervention to other stroke patients. One patient expressed disappointment when the treatment sessions came to an end.

*“I thought it was a pity that it was already over after ten times.” (P10)*

##### Experienced physical effects

Some patients reported that the rTMS treatment had improved their arm function. On the contrary, some other patients reported that they were unable to indicate a cause of their recovery. They thought that a combination of participation in the trial, rehabilitation therapies and their own willingness to recover led to improvement. Two patients noted that they did not know the difference between what to expect and what not to expect in terms of recovery (e.g. spontaneous recovery).

Some patients were unable to identify any possible improvement. Those patients did not notice a difference in arm function, because of the TMS treatment.

*“No, no not immediately. Maybe yes, of course you never know what the effect would be if you didn't do it. I have also noticed that with the medication that I take, since I’m tapering off, it suddenly gets very bad. So [the medication] did not lead to improvement, but if you do not take it, it affects you negatively. Perhaps that it also works that way for this [B-STARS intervention].” (P12)*

Some patients express the difficulty of experiencing improvement when there is no comparison.

*“I do not dare to say that. I don't know how to explain that. I got this [a stroke]. I don't know what it would be like if I hadn't done it. With or without [therapy], I don't have any comparison, so maybe it helped but maybe it did not.” (P9)*

One patient stated that it was probably too early to speak about the results: *“I think it’s too early to judge.” (P13).*

A few patients reported no (long-term) improvements in arm function following the treatment.

Often patients reported their thoughts when they had sensations in the hand muscles in response to the pulses given to the head during determination of the resting motor threshold (RMT). Those sensations often gave rise to the idea that the treatment was ‘working’. The patients were aware that the determination of the RMT was not part of the treatment, but of the set-up phase.

##### Comfort

The treatment itself was described as a relaxed experience. Patients felt comfortable and were not nervous or anxious during the treatment sessions. Two patients described their voluntary participation in the trial.

*“Yes, because otherwise you could have stopped it [treatment], if it became too much, but it didn’t.” (P39)*

The comfort of the chair where they had to sit in was highly appreciated. The chair was experienced as very relaxing and some patients almost fell asleep in it. The calm environment without too many noises, and the calmness that the researchers radiated (e.g. by talking softly) seemed to contribute to the relaxed experience that patients had in the trial. A couple of patients described the pulses given on the head as a relaxing experience. One patient described it as follows:

*“During the treatment I was very calm and strangely enough those pulses were calming and almost made me fall asleep.” (P3)*

##### Therapeutic relationship

All patients spoke highly of the research team that delivered the treatment. The researchers were seen as ‘polite’, ‘correct’, ‘friendly’ and ‘patient’. The human approach by the research team was experienced as comforting. For example, patients described that the researchers took their time in communicating with them and patients did not feel as a ‘number’. One patient was particularly pleased to receive attention that was specifically intended for him: the contact was more intimate.

Some patients expressed that they felt the researchers were grateful that they participated in the trial.

*“And I have to say you were always very friendly. Grateful. I always consider that to be important. However, it is not a decisive factor, but it is always nice when you're finished and people are happy with what you have done. If you can help someone, why not.” (P12)*

The attitude of the researchers was described as professional and trustworthy.

*“No, because you appear so confident and reliable. You know what you are talking about, in my opinion, that is very obvious. Not like: ‘maybe when we try this or that’, then I would sense doubt. But, now it is clear what we are going to do and how we are going to do it.” (P7)*

Within these narratives there is a strong sense of safety. One patient expressed appreciation for the communicated zero expectations from the researchers.

##### Receiving information

It was experienced that the research team gave clear information. Different aspects of information delivery were valued. One patient explained: *“Just good information, normal answers, calmly - they take the time to explain it.” (P2)* Another said: *“Everything was explained very neatly in regular Dutch language, the way I speak it.” (P7)* Many patients commented on the explanation of every step during the treatment sessions.

*“And they also said beforehand ‘we are going to do this now, we are doing that now, you will feel this right now.’ I also really like that.” (P13)*

Knowing what to expect was emphasized as one of the reasons for the positive information exchange.

##### Learning about the brain

The muscle sensations of the pulses had a funny component for patients and made them more aware of the working of the brain.

*“Well I didn’t find it exciting, scary or anything. Yes, really intrinsic interest in how the brain works and what is connected within your body.” (P10)*

##### No burden of added TMS treatment session

All patients perceived participation in the daily treatment sessions as an element of their daily rehabilitation program.

*“I was still in the middle of my rehabilitation process. So, then it's quite easy, like “okay it's an investigation”. At a certain point it was just scheduled in my day schedule, so it is just part of your day.” (P12)*

*“A moment of relaxation! Hahaha, in here you walk from pillar to post and sometimes you have very intensive treatments and this - this was just a tranquil moment. You didn't have to do anything myself…” (P10)*

The fact that they had a moment of relaxation and had the freedom to do nothing was appreciated by them. Regarding the number of treatment sessions, patients indicated that if there had been a need from the trial or if it would have a (more) beneficial effect, they would have participated in more sessions. Patients also saw the treatment sessions as additional therapy: *“The more [therapy], the better. Extra therapy, and just another step closer to perhaps an aid or an improvement, recovery.” (P12)* The positivity around additional therapy (implicitly) reflects the patient’s belief that ‘more therapy is better outcome’. Some patients indicated that their daily program was quite busy, which caused fatigue, however the B-STARS did not cause additional fatigue.

##### No unpleasant aspects

Overall, the patients commented favorably on the painless aspect of the treatment: *“Yes, because it didn’t hurt either. See, if it hurts then it’s different, but … it didn’t hurt.” (P4)* Next to the painless treatment, the absence of negative consequences made them feel optimistic. One patient mentioned the non-invasive aspect of the brain stimulation. The pulses were often described by the patients in a neutral way or it was indicated that you had to get used to it.

#### Concerns

##### Effects of stimulation of the brain

Some patients initially felt a bit nervous or anxious before the start of the treatment sessions. Patients were concerned about the ‘electricity’ within the brain and were unsure whether there would be negative consequences for the brain from the brain stimulation.

*“It did create some tension. Normally I am not a person who experiences tension that easily, but this created a bit of tension, because I still consider it [rTMS] as electronics, and it is my own brain. The most important part of your body, so to speak. And I was thinking: ‘I hope everything goes just right’.” (P7)*

Some patients did not completely understand the relation between the diagnostic pulses and responses in one of the hand muscles during determination of the RMT, resulting in feelings of confusion.

*“In the beginning I thought: ‘hey that stings’. You know. I could not immediately make the connection. Those shocks came and I thought ‘oooh, okay?’.” (P1)*

Others reported feelings of annoyance caused by the pulses.

*“Well at a certain point if you have to continue for longer, then you will get kind of tired of it. But it doesn't really hurt. You can feel it more clearly around your temples than on your head. But when they navigate more to your temples, then you feel it more clearly. But still it doesn't hurt or so. It's just a bit annoying. It is not really pleasant.” (P2)*

##### Equipment (chair and coil)

Most patients expressed concerns about the chair where they had to sit in during the treatment. Comfort during the treatment was experienced as important. One patient was hindered by lack of support from the head rest and arm rests being too small.

*“If I would have designed [the chair] for the person lying in it … I would have provided more support at the bottom of the neck, which makes it more comfortable. And I would make the handrail 2 centimeters wider so it is easier to place your arm on it.” (P7)*

Another patient said *“The only downside I can mention is the chair in which I had to sit. I found it quite uncomfortable to sit back and then have to tilt my head back. Because of that chair, I was sitting in such a way that it wasn’t a pleasant position, so you kind of freeze in a very uncomfortable position.” (P8)*

This patient also indicated that longer periods in the chair would not be feasible: *“So it was good that it did not last any longer, because then I would not have been able to keep up with it.” (P8)* Other patients also found the chair uncomfortable, but were not bothered by it because they were in the chair for only a short time and understood that everyone should fit in the chair.

Two patients had specific concerns regarding the coil *“Sometimes you just had an uncomfortable hairpin on your head, so sitting in the chair was a little less comfortable, but that’s all I have to say.” (P10),* and glue from the electrodes: *“I don’t like the glue … I mean not removing it, because it isn’t a band-aid. It just remains sticky for a long time.” (P5)* used during the treatment sessions.

##### Logistics

Three patients expressed that ten treatment sessions are probably enough, since no recovery would be expected beyond those sessions, because they experienced no improvements in arm function during the intervention period. Or they indicated that the uncomfortable position in the chair would be a reason to exclude additional treatment sessions. One of them would not mind to receive fewer treatment sessions: *“Shorter yes, but not longer.” (P1)* Patients experienced little or no fatigue due to the format of the therapy, but did express that the full day program of rehabilitation caused fatigue or concerns from nurses about their program.

*“The nursing staff gave me the impression that they thought that they thought it was a bit too much and a bit busy. But hey, according to me it fitted into my program and then you have to re-schedule a bit and it may mean that you had to take a shorter break occasionally.” (P10)*

### Participation in an RCT

#### General experience of recovery

The most commonly perceived changes after the treatment were improvements in arm function. In some cases, the patients described their improvements in comparison to their initial paresis or to other patients in the rehabilitation facility.

*“It [arm, body] was no longer paralyzed on the right side.” (P5)*

*“Yes! When I see people who also attend the hand therapy group, but don’t participate in the trial, I have made great progress.” (P3)*

Some patients felt that the improvements had led to noticeable impacts on daily life activities. Activities and participation seem to be major factors in patient’s lives.

*“[I] put on my socks, put on my shoes. This morning I showered myself. I just have to be careful not to slip, but I do everything by myself. Combing my hair is going okay, but this part of my hair is still difficult. And I am able to shave myself with a razor blade and shaving cream, and you see I made no cuts.” (P7)*

*“I can talk normally, I can walk, I am here by bike, I can drive, I can work, I can drink beers, hey I can basically do everything, except give people a good hand shake.” (P12)*

In most cases, the patients noted specific improvements, as a return of strength and speed, improved fine motor skills, being able to move upper extremity elements and performing better at the motor function tests.

#### Experienced psychological effects

Experienced psychological effects (i.e. benefits and concerns) of participation in an RCT were often raised by the patients, which we divided in the subthemes ‘grateful’, ‘sense of purpose’, ‘recovery as a motivator’, and ‘group allocation’.

##### Grateful

Patients had the feeling to improve, and explicitly acknowledged the power of placebo-effect.

*“Yes … When I think it has worked, then it has worked. Yes, it's that simple. You are not sure whether it has worked, but as long as you think it has worked, then it will at least have that effect.” (P8)*

It was evident in the transcripts that a number of patients felt gifted or as ‘being chosen’ when selected for the trial.

*“I said now an angel should come to help me. And the door opens … and then actually a blonde angel comes in. And she asks if I want to participate in the program. I thought that must come from God, there is no other possibility. I am serious, it really experienced it that way. So, I told my wife that I should definitely participate, because there is no other way. So, it may sound strange, because I don't go to church or anything like that, but it really felt as a gift from God to me, and if I remember I might have said it too.” (P7)*

##### Sense of purpose

Participating in the intervention gave almost all patients a sense of purpose. On one hand, patients had a sense of helping themselves, e.g. working for maximum recovery. *“I wanted it to be as soon as over, you know. And yes, I just wanted to get rid of it as quickly as possible. And yes, then the best thing I can do is by participating.” (P1)* On the other hand, patients stated that their contribution might benefit and can create a better future for other stroke patients, and they found this rewarding. *“I want to contribute to the research because it might help others. That is the only reason.” (P13)* In addition to the sense of helping oneself and others, being able to contribute to science was also purposeful for many patients.

##### Recovery as extra motivation to exercise

Regardless whether patients improved because of receiving the rTMS treatment, noticing progress works as a great motivation. Performing the motor tests periodically, as part of their participation in the clinical trial, was appreciated by the patients. It felt like a feedback moment on the progress of their recovery, allowing improvement to be noticed by comparison with the previous performance on the test.

*“So, then you notice that there is progress, I still have some progress so that also gives you motivation to continue.” (P11)*

Those quotes hint at the possibility they were more willing to put more effort in their rehabilitation than before the improvements. For some patients experiencing recovery contributed to a more positive mindset and future.

*"Yes! You get a kind of boost, because you hope that it will help. You go into your next rehabilitation [session] with a more pleasant and relaxed feeling. In your daily schedule, you might think ‘well, maybe it helps?’ You do take the positive feeling to the next one [session]." (P2)*

One patient expressed to be proud on the improvements made this far: *“And I have to admit I am very proud that it is going so well.” (P7).*

##### Disappointment and hope of group allocation

A few patients indicated that allocation to the sham group would have disappointed them, suggesting that they were hoping for personal advantage. Despite the expressed disappointment of possible allocation to the control group, they did not drop out or refuse to participate.

#### Motivation to participate

##### Personal benefit and cognitions

All patients joined the study hoping that the treatment would positively impact their arm function (recovery).

*“It is the research that you can contribute to and if you are lucky and fall into the right group, then it may turn out to be positive. That is enough motivation for me to do something. Certainly because you know that the most progress can be expected in the first three months. And it now falls within those first three months. It seems to be highly recommendable to me.” (P8)*

This patient also expressed the hope to be in the intervention group, to be able to have a chance on recovery. Most patients had the belief that the brain can be influenced from outside, for example by using non-invasive brain stimulation. One patient could not make up his mind: *“I do not know. Maybe. I do not actually know. I hope so, but I don’t know for sure.” (P4)* Different arguments were brought up why or how placebo might work.

A belief in the expertise of the researchers and the expectation that patients will not be harmed also contributed to volunteering in the study. Multiple patients felt that “*If you start with something, you need to finish it” (P9)* which ensured constant motivation during the trial.

Several patients expressed that participation was an offer too good to refuse. *“If you get the chance to do something like this and you can help someone forward with it, I take that chance. It doesn’t cost a dime! Take that chance.” (P7)* Linked to this, patients also felt motivated by the following beliefs: “*It doesn’t hurt to try”* and “*Nothing ventured, nothing gained.”* The concept of karma was also described by one of the patients: *“You always get it [friendliness] in return if you act friendly.” (P7).*

Most patients described that their attitude towards rehabilitation and recovery is of importance for the outcome.

*“I tried to stay reasonably positive. Because yes it was made clear, that the way you look at things, determines what your recovery will look like.” (P12)*

*"But if you immediately say no, then nothing happens either … " (P4)*

A positive mindset and openness to experience seem ingredients for a fruitful recovery according to those patients.

Undergoing the treatment provided patients with an opportunity to be involved in scientific research, which they find fascinating. Their curiosity was fed.

##### Altruism

One of the most common reasons for participation in the trial was the hope to help current and future stroke patients. Several patients explicitly described their participation might benefit offspring or grandchildren.

*“Well, I hope that I can offer my children and granddaughter a longer life through this. Maybe … the benefit, that when having a stroke, or whatever you call it, that intervention is possible in a different way than what is now. Because I saw it as extra therapy and I have done science a service and I hope to my offspring too. I am happy that I have been able to contribute to the research; for my offspring and the rest of the people.” (P5)*

This quote reflects the prevailing norms and values that are of importance to that person. Patients also demonstrated the willingness to participate to the advancement of science. Participation in the intervention provided patients with an opportunity to actively contribute to scientific research. One patient gave the example that participating would increase the diversity of age in the trial:

*“… most people with whom I have been here are 60+. So, I thought maybe it is good, or interesting or nice for you … that also a younger person, I was 30 when it happened. That you can also see what it does to a young person. Perhaps there were very different results with me than with a fellow patient who is already in his seventies or sixties.” (P12)*

## Discussion

The aim of this qualitative study was to gain insight into how rTMS treatment for upper limb recovery within stroke rehabilitation was experienced by patients who participated in the clinical trial. Overall, the patients reported positive experiences with the TMS treatment and believed that receiving rTMS had benefited their arm function. Aspects of the treatment that were experienced most favorably were the comfort of the treatment setting (e.g. moment of relaxation) and the absence of pain and side-effects. The main concerns were the fear of or uncertainty about negative consequences from the electrical currents within the brain and the annoyance of the pulses on the head. Most patients participated in the study to contribute to knowledge for treatment of future stroke patients and for personal benefit. Participation in a clinical trial was also experienced as a grateful experience and gave patients a sense of purpose.

### Benefits and concerns related to rTMS treatment

Patients felt that the rTMS treatment was effective for the recovery of their affected arm, even though they were blinded from the treatment assignment –which could be sham stimulation– and outcome. Effectiveness was described by patients as being able to perform fine and gross movements with the affected arm (i.e. reduction of impairment and disability), being able to wash and dress themselves, and being able to participate in traffic as cyclist or car driver (i.e. activity and participation). These outcomes are contrary to the only (known) previous qualitative study in the field of non-invasive brain stimulation of Triccas et al. (2018) [14], where patients felt that receiving tDCS and robotic therapy was especially effective for their strength and tightness in the affected upper limb. This discrepancy in results could be explained by the design of the studies. Triccas et al. (2018) [14] used a robot, paired with tDCS, which trains hand grip rather than hand movements. The lack of ability to carry out two-handed tasks easier was one of the main concerns reported by their patients. The expressed hope and expectations of personal benefit for recovery are consistent with the main reasons for participation stated by patients in our trial. Assumptions of therapeutic benefit from participation in (stroke rehabilitation) research has also been found and discussed in other studies [27, 28].

The included patients evaluated receiving rTMS treatment positively. Positive experiences arose from the comfortable treatment settings, the interaction with the research-therapists, the way of information delivery, learning experiences, and the absence of pain or negative consequences. Comfort of the treatment was related to the way the treatment was built into their daily schedule, the low burden of the treatment procedure (i.e. maximally five minutes; comfortable position in the treatment chair), and the setting in which they received the brain stimulation. Patients were pleased that the treatments were scheduled and they experienced the treatments as a moment of relaxation during their full and sometimes tiring daily rehabilitation program. The short duration of the treatments may have ensured that the patients did not experience the intervention as a burden. However, in a qualitative descriptive study a high intensity programme was not viewed as a barrier to engagement for the included stroke patients. The acceptability and engagement with the high intensity programme was mediated by several factors, including making progress, internal and external motivators, and other group members [29]. The treatment room in our study, which was experienced as quiet and low on stimuli, also contributed to the relaxed experience. The importance of comfort has also been emphasized by patients who evaluated novel stroke technologies [30].

The interaction with research staff administering the treatment was highly valued among the patients. Attributes of the researchers, i.e. being calm, competent, transparent and respectful, enabled a sense of safety and a strong therapeutic relationship. The friendly attitude of the research staff was highly valued and made patients look forward to their treatment session. These findings agree with results from a qualitative study [31] that reported that to be treated with respect and dignity was the core factor contributing to elderly stroke patients’ satisfaction with rehabilitation therapy. Subcategories as being treated with humanity, having confidence and trust in professionals, and dialogue and exchange of information were other determinants of satisfaction [31]. In our study, the clarity of information and the step-by-step explanation of the different actions ensured that patients knew what to expect, and made them feel safe. The desire for information has previously been recognized as a key coping strategy and as a resource for psychosocial adjustment and empowerment [31, 32].

However, in addition to reported feelings of therapeutic benefit and positive experiences of undergoing rTMS treatment, some concerns were also identified. One of the main concerns were feelings of anxiety about the ‘electricity’ within the brain and uncertainty about possible negative consequences. This finding is consistent with findings from Triccas et al. (2018) [14] who reported that patients undergoing tDCS treatment had concerns about the ‘electricity’ applied via the electrodes, and were insecure about possible negative consequences. Some patients found the stimulation pulses annoying. The somatic scalp sensation due to TMS-induced activation of superficial nerves or muscles might feel different for each person [33] The reported annoyance of the TMS pulses, however, appear less severe compared to the reported itchy, painful, and burning sensations after tDCS [14]. The therapeutic potential of TMS is not limited to stroke patients, as efficacy, tolerability and safety have also been demonstrated for other neurological and psychiatric disorders [9]. TMS has been clinically approved for treatment of major depressive disorder, for which there are comprehensive guides for the safe administration of TMS while ensuring patient comfort (e.g. with regard to head support or cushioning lighting and room temperature, and small adjustments in the placement or rotation of the coil) [34].

### Benefits and concerns related to participation in an RCT

The beneficial psychological impact of trial participation, beyond those of the treatment, has been increasingly recognized [35, 36]. Patients who perceived recovery during participation in our trial found this hopeful and motivating, which made them more likely to put effort in their rehabilitation. Our findings are consistent with data from studies that investigated stroke survivors’ perspective of upper limb recovery after stroke [37–42]. An individual’s experience of recovery helps to maintain their motivation [37, 38]. Furthermore, the hope for and belief in further recovery is related to an individual’s attempt to maximize upper limb recovery (i.e. ‘keeping doors open’) and overall stroke recovery [39, 41, 42].

Patients reported a powerful belief that they might receive an effective treatment, and acknowledged the possible effects on health and well-being. Expectancy, what a patient thinks or expects to happen as a result of the treatment, is one of the many contributing factors to the recognized placebo response [43]. In addition to a placebo effect, being offered or ‘chosen’ to participate in a trial, resulting in a sense of hope, might contributed positively to emotional well-being. Trial participation as a privilege has also been reported by patients enrolled in a cancer-related clinical drug treatment trial [44]. In our study, trial participation was also associated with the satisfaction of feeling useful and having a sense of purpose. Those feelings derived from being able to actively contribute to one’s recovery process, contributing to help for current and future stroke patients, and being part of scientific research resulting in more knowledge and therapeutic advancements. Curiosity and interest in scientific research were also mentioned as reasons to participate. By participating in the intervention, patients could learn more about the (working of the) brain and experience a novel rehabilitation technique. The societal benefits of participating in a trial and contributing to something greater than yourself, which may be consistent with existential well-being, has been reported for patients before [35, 45]. The concept of “intergenerational altruism” has been introduced to describe altruistic motivations to participate in research for the benefit of younger generations [46].

Some patients shared concerns about allocation to the control group, which could limit their chance of additional improvement. Several studies have explored the psychological burden for patients participating in an RCT, who were informed by the result of randomization, and acknowledged the discomfort of being randomized, and anxiousness or embarrassment to receive placebo [27, 47]. However, in our study the patients were unaware of group allocation. The concept of random allocation might be more difficult to accept for patients who know that they are receiving placebo, because they can compare themselves to the non-placebo group.

### Limitations

Our study has some limitations. First, the interviews were not conducted at a fixed time after the intervention period, causing some patients to have to recall memories from one to 2 years ago. Difficulties in recalling experiences could introduce a risk of cognitive bias. However, even in patients who were interviewed one to 2 years after participation in the trial, we noticed that they could reproduce their memories, suggesting that a period to process and reflect upon your experiences could also lead to a more matured memory. Second, the first author designed the protocol, recruited patients for the RCT, and analyzed the qualitative data, introducing the risk of bias. To minimize this risk, the author (LJ) who undertook the interviews and took no part in the RCT, also assisted in the data analysis. Third, although representative for many European hospitals, the rehabilitation setting in which our study was conducted may be different from other healthcare situations.

### Clinical implications and directions for future research

Several treatment characteristics that could lead to effective implementation of rTMS in clinical care and implications of the findings for inpatient stroke rehabilitation and recommendations for future rTMS trials were identified from the interviews.

Implementation in clinical care.

According to the experiences of the patients in an inpatient facility it is feasible to add rTMS therapy to a daily inpatient stroke rehabilitation program. Undergoing this treatment was not experienced as a burden. Careful communication seems to be a key factor in a patient’s sense of safety and satisfaction with rTMS treatment participation. Transparency, explanation of the procedures in layman’s terms, and a humane approach to the patient seem to be important factors. To anticipate to the patient’s concerns about electrical currents in the brain, negative consequences and painful sensations, personnel should pay attention to recurrently explaining the mode of action of rTMS, the non-invasive character of the treatment, and the limited possible risks. It is essential to pay close attention to the settings (i.e. equipment use, support materials, environment) in which the treatment is delivered to prevent discomfort. The active involvement of patient’s perspectives in the design of rTMS treatment delivery could be central in this.

#### Inpatient stroke rehabilitation

In the present study, we identified that patients value a moment of relaxation in between appointments of their rehabilitation program. These moments of rest could be built into the standard routine of inpatient stroke rehabilitation. According to the patients, the relaxation was felt because they could sit in a comfortable declined chair in a quiet room, and had the freedom to do nothing. The full procedure of preparation, installation and treatment delivery took a maximum of 10 min (the actual rTMS treatment lasted only 40 s), showing that this effect can be achieved in a relatively short period of time.

We also found that patient value feedback on their progress of motor recovery, as it contributes to an individual’s expectation, motivation, and attempts to maximize upper limb recovery. This could include communicating repeated measurements to the patient in advance and the use of patient reported outcome measures (PROMs). Repeating measurements over time and at specific time-points post-stroke (i.e. at least 3 months stroke, according to the SRRR [48]) is relevant for clinical care as research.

#### Future rTMS trials

Our results emphasize that participation in scientific research makes people feel useful and empowers them to contribute to something bigger than themselves. Future rTMS trials should consider describing the positive effects of participation in scientific research in their patient information letter. In addition, involved stakeholders (e.g. nursing staff, facility personnel) should also be aware of the positive effects that patients can experience in trial participation, especially when there are doubts about the feasibility of a study in rehabilitation care. The patient information letter should also clearly explain the working of the TMS and the possible side-effects.

## Conclusions

The present qualitative study provides critical insights in how the design and delivery of rTMS treatment for upper limb recovery is experienced by patients. The acquired information can aid in improvement of the design of future rTMS trials and implementation in routine stroke rehabilitation programs. rTMS was well accepted and even enjoyed by patients. Comfortable treatment settings, respectful communication, and transparent information contributed to the experienced satisfaction. The experiences and preferences of stroke patients could be useful in the design of future rTMS studies and the implementation of rTMS in clinical care.

## Supplementary information

**Additional file 1.**

**Additional file 2.**

**Additional file 3.**

## Data Availability

The datasets generated and analysed during the current study are not publicly available due to privacy reasons of patients, but are available from the corresponding author on reasonable request.

## Abbreviations

ADL: Activities for daily living
B-STARS: Brain-STimulation for Arm Recovery after Stroke
COREQ: Criteria for reporting qualitative research
cTBS: Continuous theta burst stimulation
M1: Primary motor cortex
NIBS: Non-invasive brain stimulation
RCT: Randomized controlled trial
rTMS: Repetitive transcranial magnetic stimulation
tDCS: Transcranial direct current stimulation
TMS: Transcranial magnetic stimulation
