# Supplementary material for: Exploring the experiences of stroke patients treated with transcranial magnetic stimulation for upper limb recovery: a qualitative study
Source: BMC Neurol. 2020 Oct 6;20:365. doi: 10.1186/s12883-020-01936-5 (PMC7541313; doi:10.1186/s12883-020-01936-5)
Supplement: Supplementary file 2 — Additional file 2. [file 12883_2020_1936_MOESM2_ESM.docx]

Additional file 2. Interview guidelines

Topics interviews

- *General experience with the brain stimulation treatments*
- *Perceived pleasant aspects of the treatment*
- *Perceived unpleasant aspects of the treatment*
- *Motivation for participating and completing the program*
- *Emotions experienced by the participant during different stages of the program:*
  - *Before start of the treatments*
  - *During the treatments*
  - *As the treatment progress*
- *Experiences with the different sensations/elements of the treatments:*
  - *Coil*
  - *Electrodes*
  - *Wrist band*
  - *Pulses*
  - *Chair*
  - *Physical sensations*
- *Perceived benefits of the program*
  - *Effectivity of the treatments*
  - *How are the participants since they completed their treatments?*
- *Perceived disadvantages of the program*
- *Duration of the treatments*
- *Recommendation to other stroke patients*
- *Experiences with the arm training*
- *Experiences with information from the research(ers):*
  - *Before start of the treatments*
  - *During the treatments*
  - *Clarity of the information*
  - *Understanding what might be expected from the participants*
- *General cognitions of the participants:*
  - *Expectations/cognitions about brain stimulation before start treatments*
  - *Expectations/cognitions about brain stimulation after completing the treatments*
  - *Opinions about the developments of new treatment techniques*
  - *Working of the brain*
- *Thoughts about how the treatments fit in their regular program*
- *Intensity of participation*
- *Points of improvements from the participants*
